# Supplementary material for: Regional intergenerational mobility and corporate innovation: Evidence from China
Source: PLoS One. 2023 Apr 3;18(4):e0283588. doi: 10.1371/journal.pone.0283588 (PMC10069787; doi:10.1371/journal.pone.0283588)
Supplement: S1 File — Appendix A. Classification of academic qualifications. Appendix B. Cultural indicators of each province. Appendix C. Market_oriented reform indicators of each province in 2014. (DOCX) [file pone.0283588.s001.docx]

**Appendix A.**

Classification of academic qualifications

| **Educational attainment level** | **Schooling years (assignment in this study)** |
| --- | --- |
| **Illiteracy** | 0 |
| **Primary school** | 6 |
| **Junior high school** | 9 |
| **Senior high school** | 12 |
| **Associate degree** | 15 |
| **Bachelor degree** | 16 |
| **Master or Doctor degree** | 19 |

Note: Our classification criteria are in accordance with China’s educational regulations.

**Appendix B.**

Cultural indicators of each province.

| **Province** | **Future orientation** | **Performance orientation** | **Province** | **Future orientation** | **Performance orientation** |
| --- | --- | --- | --- | --- | --- |
| Beijing | 4.17 | 4.60 | Shandong | 4.25 | 4.61 |
| Tianjin | 4.22 | 4.77 | Henan | 4.23 | 4.70 |
| Hebei | 4.22 | 4.67 | Hubei | 4.21 | 4.88 |
| Shanxi | 4.25 | 4.76 | Hunan | 4.39 | 4.91 |
| Inner-Mongolia | 4.09 | 4.64 | Guangdong | 4.37 | 4.66 |
| Liaoning | 4.17 | 4.53 | Guangxi | 4.35 | 4.67 |
| Jilin | 4.26 | 4.78 | Sichuan | 4.04 | 4.42 |
| Heilongjiang | 4.22 | 4.65 | Guizhou | 4.15 | 4.86 |
| Shanghai | 4.26 | 4.51 | Yunnan | 4.11 | 4.73 |
| Jiangsu | 4.17 | 4.60 | Shaanxi | 4.07 | 4.51 |
| Zhejiang | 4.23 | 4.69 | Gansu | 4.20 | 4.60 |
| Anhui | 4.32 | 4.75 | Qinghai | 4.17 | 4.49 |
| Fujian | 4.24 | 4.64 | Ningxia | 4.37 | 4.87 |
| Jiangxi | 4.34 | 4.89 | Chongqing | 4.20 | 4.78 |

Note: As the sample cities do not include Hainan, Tibet, and Xinjiang, the data shows only 27 provinces and municipalities.

**Appendix C**

Market_oriented reform indicators of each province in 2014.

| **Province** | **Market1** | **Market2** | **Province** | **Market1** | **Market2** |
| --- | --- | --- | --- | --- | --- |
| Beijing | 7.30 | 4.78 | Shandong | 9.64 | 9.59 |
| Tianjin | 8.43 | 8.04 | Henan | 9.37 | 8.80 |
| Hebei | 8.26 | 6.60 | Hubei | 8.50 | 8.53 |
| Shanxi | 5.31 | 7.45 | Hunan | 8.43 | 8.97 |
| Inner-Mongolia | 6.90 | 8.15 | Guangdong | 9.99 | 9.57 |
| Liaoning | 8.45 | 8.46 | Guangxi | 7.41 | 9.14 |
| Jilin | 7.45 | 8.32 | Sichuan | 7.31 | 8.09 |
| Heilongjiang | 5.39 | 7.96 | Guizhou | 4.57 | 8.39 |
| Shanghai | 8.53 | 8.42 | Yunnan | 5.52 | 6.81 |
| Jiangsu | 10.34 | 8.25 | Shaanxi | 5.00 | 7.52 |
| Zhejiang | 9.88 | 8.26 | Gansu | 3.28 | 7.32 |
| Anhui | 8.65 | 8.50 | Qinghai | 4.59 | 1.46 |
| Fujian | 9.40 | 9.73 | Ningxia | 6.45 | 7.45 |
| Jiangxi | 8.68 | 9.14 | Chongqing | 8.50 | 8.02 |

Note: (1) The data are taken from the Chinese Research Data Services (CNRDS) Platform. (2) As the sample cities do not include Hainan, Tibet, and Xinjiang, the data shows only 27 provinces and municipalities.
