# Supplementary material for: Regional intergenerational mobility and corporate innovation: Evidence from China
Source: PLoS One. 2023 Apr 3;18(4):e0283588. doi: 10.1371/journal.pone.0283588 (PMC10069787; doi:10.1371/journal.pone.0283588)
Supplement: Attachment — Submitted filename: Response to Reviewers.docx [file pone.0283588.s002.docx]

**Response to Reviewers**

**Dear Reviewers:**

First of all, please allow us to take this opportunity to express our heartfelt thanks to you for taking time out from your busy schedule to review this manuscript. You have provided us with constructive comments and suggestions, which are of great help for us to further improve this manuscript. We have carefully reviewed and revised the manuscript according to your valuable comments and suggestions. Here, we explain the revised work in detail below and provide the point-by-point responses to the reviewers’ comments.

**Respond to the Reviewer #1**:

*This paper examines the impact of regional intergenerational mobility on corporate innovation based on the data from China Labor-force Dynamic Survey (CLDS) and A-share listed companies. They find that regional intergenerational mobility promotes corporate innovation both quantitatively and qualitatively, and government-enterprise human capital allocation and fairness perception are the two channels. I recommend that the paper can be accepted by the journal after minor revision. Before the publication, several errors should be corrected by the authors.*

1. *The introduction needs to be adjusted. Specifically, lines 66-77 in the original text need to be adjusted to the end of the text. Generally speaking, in the introduction, you only need to introduce the background and highlight the innovation of the article, without presenting the research results.*

**Response:** We thank the reviewer for bring our attention to these inspiring works. In the revised manuscript, we have removed the content you mentioned.

*2. The research literature review is slightly weak and needs to be updated to 2022. More support is given in the literature review section.*

**Response:** Thank you for your comments. According to your suggestion,we have added 5 more pieces of literature in the corporate innovation and further deepened the discussion in the revised manuscript.

“Enterprise resources are divided into tangible resources (e.g., raw materials, machinery and equipment) and intangible resources (e.g., reputation, environment). From the perspective of resource dependence, Hart(1995) demonstrated that the environment of an enterprise is an important indicator of comparative advantage. Without the financial and environmental resources required for successful innovation activities, it is difficult for enterprises to achieve a high level of innovation output (Khan et al., 2020; Yu, et al., 2022;) . In addition, stakeholder theory holds that corporate activities are closely connected with and even influenced by various stakeholders (including employees, customers and society) (Wen et al, 2022; Ullah et al., 2022;).”

**Added references for question 2 of Reviewer 1 (All of them are added in the revised manuscript):**

Hart, S.L. A natural-resource-based view of the firm, Academy of Management Review, 1995, 20, 4, 986-1014. https://10.2307/258963

Khan, Z., Hussain, M., Shahbaz, M., Yang, S., Jiao, Z. Natural resource abundance, technological innovation, and human capital nexus with financial development: a case study of China, Resource Policy, 2020, 65, 101585. https://10.1016/j.resourpol.2020.101585

Yu, XJ, Liao, J, Smith, D. How does regional religiosity influence corporate innovation? New evidence from China, Applied Economic Letters. 2022. https://10.1080/13504851.2022.2152416

Wen, HW, Lee, CC, Zhou, FX. How does fiscal policy uncertainty affect corporate innovation investment? Evidence from China’s new energy industry. Energy Economics. 2022, 105, 105767. https://10.1016/j.eneco.2021.105767

Ullah, F, Jiang, P, Elamer, AA, Owusu, A. Environmental performance and corporate innovation in China: The moderating impact of firm ownership, Technology Forecasting and Social Change. 2022, 184, 121990. https://doi.org/10.1016/j.techfore.2022.121990

*3. “firm”, "Company" and “enterprise” need more accurate expression, or unified expression.*

**Response:** Many thanks for your constructive suggestions. In addition to some fixed collocations, such as firm-level, we have unified other words in the revised manuscript.

*4. In the part of Empirical Results and analyzes, it is suggested to directly use variable meanings rather than abbreviations, which is more convenient for readers to understand. For example: Abmobility25, Hcapital, etc.*

**Response:** Thanks to the reviewer’s useful suggestions. Following the reviewer’s suggestion, we have replaced the abbreviations with variable meanings in the revised manuscript, which should help readers better understand our study.

*5. Set the tables according to journal guidelines.*

**Response:** We thank the reviewer for raising our awareness of this issue. In the revised manuscript, we have made corrections.

*6. The authors are advised to add the limitations of this study and possible future research directions after the conclusions and policy implications.*

**Response:** Thank you for your constructive suggestion. We have provided the supplement in **Section Conclusion and policy implications**.

“There are some limitations to this study. Firstly, referring to the existing literature, we try to measure the regional intergenerational mobility. Due to the limitation of data acquisition, only cross-sectional proxy variables can be obtained in this paper. Although there is a lot of evidence that intergenerational mobility at the city level is relatively stable in the short term, we cannot perfectly solve the doubts caused by non-temporal variables. Secondly, the research sample of this study is limited to Chinese listed companies that need to be strictly regulated. Among industrial enterprises above designated size, the proportion of listed companies is relatively small. If future studies extend the sample to all industrial enterprises above size, other findings may be possible. Finally, we also can compare whether the impact of regional intergenerational mobility on corporate innovation is different between OECD countries and emerging countries.”

**Response to the Reviewer#2**:

*Based on the title of the manuscript, it looks promising and can be an insightful study. However, there are some problems to be further improved as well. Please consider the following points to improve your work.*

*1.In the abstract section, I note that regional intergenerational mobility has a positive impact on both quantity and quality of firm innovation. However, the authors do not explain this in section3.2.1. Detailed information is required.*
**Response:** Following the reviewer’s suggestion, in the revised manuscript we have provided more explanation on the difference between innovation quantity and innovation quality.

*“*According to the rules of Chinese patent law, invention patents belong to the high level of innovation and are generally regarded as a proxy variable of innovation quality. We simultaneously apply the total number of patent applications to represent the amount of enterprise innovation.*”*

*2.In Section 2.1, the authors also discuss the possible negative relationship between high regional intergenerational mobility and firm innovation. I think the hypothesis of H1B is not reliable, and it is not convenient for readers to understand the paper clearly. Therefore, I suggest this hypothesis can be removed.*
**Response:** Thanks to the reviewer’s constructive suggestions. We have made a correction and removed the hypothesis (H1B) mentioned by the reviewer in the revised manuscript.

*3.In Section 2.3, I suggest that the authors should be more detailed in their theoretical analysis.*
**Response:** We thank the reviewer’s helpful comments. In the revised manuscript, we have added more theoretical analysis. For more details, please see the **Section Reigonal intergenerational mobility and corporate innovation**, and **Section Innovation demand side** in the revised manuscript.

*4.In Section 3.2.2, the authors should explain why absolute intergenerational mobility is chosen as the proxy variable for regional intergenerational mobility.*
**Response**: Thanks you for your constructive suggestion. In the revised manuscript, We have provided reasons for using absolute mobility to characterize regional intergenerational mobility in this paper.

“Intergenerational mobility is usually divided into relative mobility and absolute mobility. Absolute mobility refers to the expected achievement of children given their parents’ income levels. By contrast, relative mobility focuses on the relative differences between children from different family backgrounds. However, it is important to point out that the increase in relative mobility is probably result from the fact that outcomes for children from low-income families get worse. In such circumstances, higher relative mobility is not necessarily desirable. We, thus, use absolute mobility as a proxy variable for regional intergenerational mobility.”

*5. In lines 281-282, for the sentence “Besides, we also controls the fixed effect of years and industries.”, the pronoun “we” must be used with a non-third-person form of a verb “control”.*
**Response**: We thank the reviewer for raising our awareness of this issue. In the revised manuscript, we have made corrections.

*6.In line 309, it should be “As we can see”.*
**Response**: Thanks to the reviewer’s comment. We have corrected the grammatical error in the revised manuscript.

*7. In line 332, there is a spelling mistake in the word “obtanined”.*

**Response**: Thanks for pointing out our writing slip. According to the reviewer’s useful suggestion, we have made correction.

**Response to the Reviewer#3**:

*This paper investigates the impact and mechanism of regional intergenerational mobility on corporate innovation. Higher regional intergenerational mobility is found to have a significant positive impact on the innovative activities of local firms. This paper proposes the possible mechanisms for this finding, such as human capital allocation and fairness perception. This is an interesting topic and I have the following comments.*

*1.The authors need to add a sentence explaining the proposed methodology. Keywords must be written in alphabetical order.*
**Response**: Following the suggestion by the reviewer, in the revised manuscript we provide supplements and amendments.

*2.The authors stated that this study follows a prior study that measures regional intergenerational mobility at the city level. However, they need to explain why this study should measure regional intergenerational mobility at the city level.*
**Response**: Thank you for your comments. We agree that your comments are very insightful and constructive. Therefore, we have made the following revisions in the revised manuscript.

“Moreover, some scholars have found significant differences in intergenerational mobility among American states. To improve the accuracy of our research, we need to pay attention to intergenerational mobility at the city level.”

*3.Significance (Table 2 and following) is not a %, but a number: 0.01, 0.05 etc.*
**Response**: We thank the reviewer for raising our awareness of this issue. In the revised manuscript, we have made corrections.

*4.The authors should add the study limitations and suggestions for future studies.*

**Response**: Thank you for your constructive suggestion. We have provided the supplement in **Section Conclusion and policy implications**.

“There are some limitations to this study. Firstly, referring to the existing literature, we try to measure the regional intergenerational mobility. Due to the limitation of data acquisition, only cross-sectional proxy variables can be obtained in this paper. Although there is a lot of evidence that intergenerational mobility at the city level is relatively stable in the short term, we cannot perfectly solve the doubts caused by non-temporal variables. Secondly, the research sample of this study is limited to Chinese listed companies that need to be strictly regulated. Among industrial enterprises above designated size, the proportion of listed companies is relatively small. If future studies extend the sample to all industrial enterprises above size, other findings may be possible. Finally, we also can compare whether the impact of regional intergenerational mobility on corporate innovation is different between OECD countries and emerging countries.”
